# Supplementary material for: Concomitant immunity to M. tuberculosis infection
Source: Sci Rep. 2022 Dec 1;12:20731. doi: 10.1038/s41598-022-24516-8 (PMC9713124; doi:10.1038/s41598-022-24516-8)
Supplement: Supplementary file 1 — Supplementary Information. [file 41598_2022_24516_MOESM1_ESM.docx]

**Supplementary Material**

**All data generated or analyzed during this study are included in this published article and are available upon request.**

*HostSim Equations with the addition of Resident Memory T cells*

The *HostSim* granuloma model equations are shown in full detail in previously published work (42). Briefly, the system of 20 ordinary differential equations captures intracellular and extracellular bacterial numbers, various macrophages, various T cells and pro- and anti-inflammatory cytokines across time. In this work, we add a resident memory T cell population (Trm) to reinfection granulomas:

$$T_{rm}= T_{rm}(0)e^{d_{Trm}t}$$

where $d_{TRM}$is the death rate of Trm and is assigned as 0.03, 0.0012, or 0.0001 cells/day, depending on the study. $T_{rm}(0)$ = [1-10] and is sampled according to a Latin Hypercube sampling scheme (56), like other parameters in *HostSim*. All other equations remain unchanged, except intracellular bacteria, which now includes a term from interactions between macrophages and Trm cells that leads to intracellular bacterial death:

$$\frac{dB_{I}}{dt}=\alpha_{19}\frac{B_{I}}{M_{I}}M_{I}\left( 1-\frac{\frac{B_{I}}{M_{I}}}{N} \right)+k_{2}\frac{N}{2}M_{R}\left( \frac{B_{E}}{B_{E}+c_{9}} \right)-k_{17}NM_{I}\left( \frac{B_{I}^{2}}{B_{I}^{2}+N^{2}M_{I}^{2}} \right)-k_{14a}\frac{B_{I}}{M_{I}}M_{I}\left( \frac{\left( \frac{T_{C}+w_{3}T_{1}}{M_{I}} \right)}{\left( \frac{T_{C}+w_{3}T_{1}}{M_{I}} \right)+c_{4}} \right)-k_{14b}\frac{B_{I}}{M_{I}}M_{I}\left( \frac{F_{\alpha}}{F_{\alpha}+f_{9}I_{10}+s_{4b}} \right)-k_{52}\frac{B_{I}}{M_{I}}M_{I}\left( \frac{\left( \frac{\frac{T_{C}}{B_{i}+1}\left( \frac{T_{1}}{T_{1}+c_{T_{1}}} \right)+w_{1}T_{1}}{M_{I}} \right)}{\left( \frac{\frac{T_{C}}{B_{i}+1}\left( \frac{T_{1}}{T_{1}+c_{T_{1}}} \right)+w_{1}T_{1}}{M_{I}} \right)+c_{52}} \right)-\mu_{B_{I}}B_{I}+\mu_{M_{I}}\frac{B_{I}}{M_{I}}M_{I}- k_{BIdeathTRM}*M_{I}*T_{rm}*\frac{B_{I}}{M_{I}}$$

Where $k_{BIdeathTRM}$ = [0.3, 0.8] bacteria/day consistent with rate constants of intracellular bacteria death from interactions with other T cells in *HostSim*, and identified via manual tuning to match the reinfection CFU dynamics of the NHP study by Cadena et al (29).


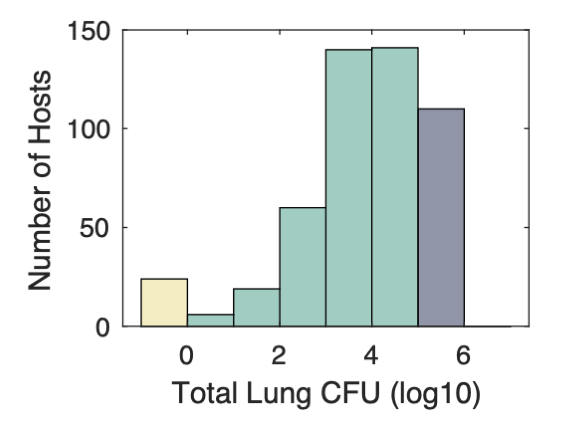


**Supplementary Figure S1: Histogram of Total Lung CFU in *HostSim* virtual population of 500 hosts.** Total lung CFU calculated by summing CFU across all granulomas in a host at day 200 following primary infection. Yellow represents hosts that are classified as Mtb eliminators (total Lung CFU < 1), dark blue represents hosts classified as active TB cases (total Lung CFU > 10^5^) and green represents latently infected individuals (LTBI), i.e. those that control infection. Across a population of 500 hosts, 110 are classified as active TB cases, 366 are classified as LTBI, and 24 are classified as Mtb eliminators. This breakdown of responses across a virtual population of 500 individuals represents our expected outcomes when calculating reduced risks of developing active TB following reinfection.


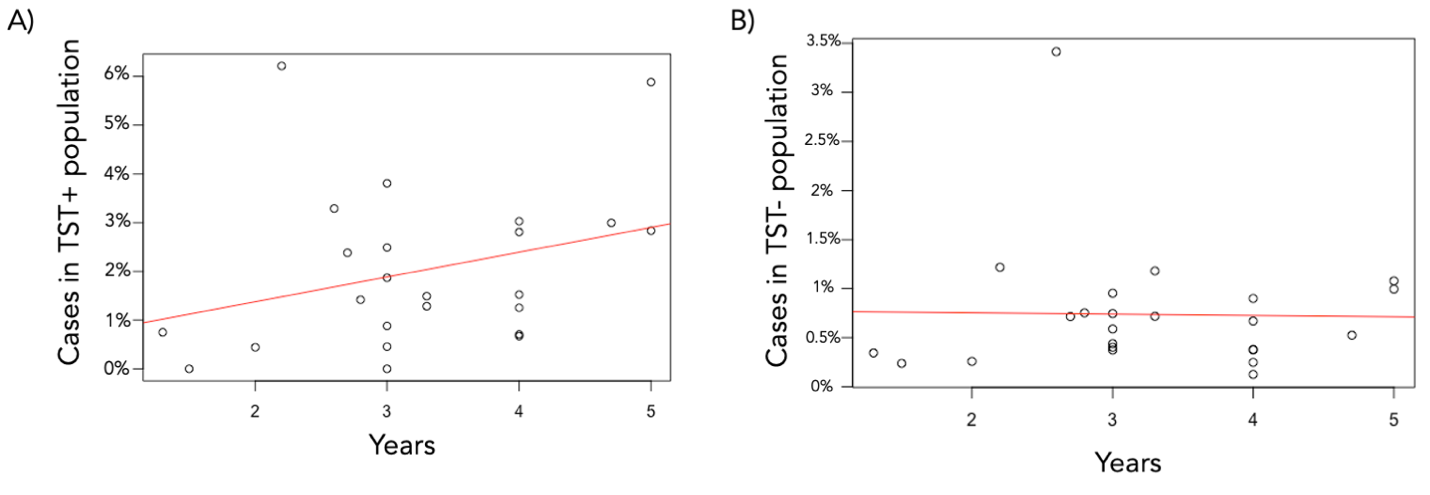


**Supplementary Figure S2: Percentages of active TB cases per study for TST+/TST- individuals prior to the study.** A) The percentage of TST+ individuals who develop active TB across time in the meta-analysis by Andrews et al. (22). B) the percentage of TST- individuals who develop active TB across time. Each data point is a single study, where the x-axis represents the length of the study. Note, studies with a longer period of observation (closer to 5 years along the x-axis) display greater percentages of active TB cases among the TST+ population compared to those where students were observed for two years or less. As shown by the red trend lines in both the TST+ and TST- populations, this suggests that active TB reinfection cases tend to occur at later time points following initial infection. This association is not statistically significant, but the greater percentage of active TB cases during longer studies suggests a potentially waning protection from concomitant immunity.


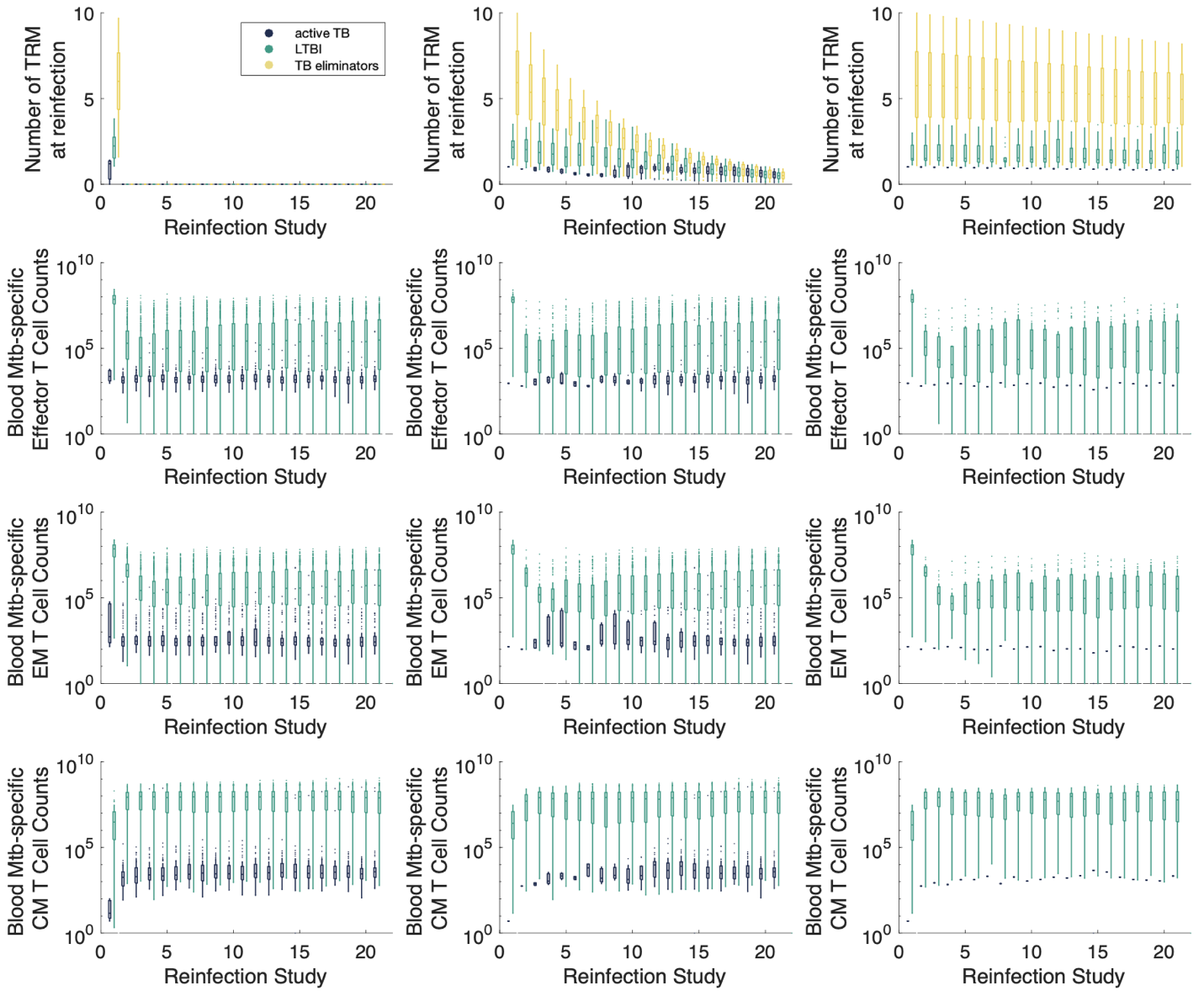


**Supplementary Figure S3: Blood T cell counts delineate active TB and LTBI cases for each set of reinfection studies.** Box-and-whisker plots show the distribution of Trm, Mtb-specific effector, effector memory or central memory T cells in the blood for hosts that were active TB, LTBI or Mtb eliminators following reinfection. Dark blue=active TB, Green = LTBI, yellow=Mtb eliminator. Each column represents each set of the three reinfection studies; where the death rate of Trm cells from left to right is *d_Trm_* =0.03, *d_Trm_* =0.0012, and *d_Trm_* =0.0001 cells/day.
